# Priorities when deciding on participation in early-phase gene therapy trials for Duchenne muscular dystrophy: a best–worst scaling experiment in caregivers and adult patients

**DOI:** 10.1186/s13023-019-1069-6

**Published:** 2019-05-09

**Authors:** Ryan S. Paquin, Ryan Fischer, Carol Mansfield, Brennan Mange, Katherine Beaverson, Annie Ganot, Amy Strong Martin, Carl Morris, Colin Rensch, Valeria Ricotti, Leo J. Russo, Alesia Sadosky, Edward C. Smith, Holly L. Peay

**Affiliations:** 1 RTI International, Research Triangle Park, North Carolina USA; 2 Parent Project Muscular Dystrophy, Hackensack, NJ USA; 3 RTI Health Solutions, Research Triangle Park, North Carolina USA; 4 Pfizer, Inc, New York, NY USA; 5 Solid Biosciences, Cambridge, MA USA; 6 UCLA, California, Los Angeles USA; 7 Duke University, Durham, North Carolina USA

**Keywords:** Gene therapy, Duchenne muscular dystrophy, Stated preference, Best-worst scaling

## Abstract

**Purpose:**

Several gene therapy trials for Duchenne muscular dystrophy initiated in 2018. Trial decision making is complicated by non-curative, time-limited benefits; the progressive, fatal course; and high unmet needs. Here, caregivers and patients prioritize factors influencing decision making regarding participation in early-phase gene therapy trials.

**Methods:**

We conducted a best-worst scaling experiment among U.S. caregivers and adults with Duchenne (*N* = 274). Participants completed 11 choice sets, choosing features they cared about most and least when deciding whether to participate in a hypothetical gene therapy trial. We analyzed the data using sequential conditional logistic regression.

**Results:**

Participants prioritized improved muscle function in trial decision making. Concerns about participation limiting later use of gene transfer and editing were also important, as were improved lung and heart function. Low risk of death fell near the middle. Participants cared least about muscle biopsies and potential for randomization to placebo. Adults with Duchenne and caregivers of non-ambulatory children significantly prioritized improved lung function compared to caregivers of ambulatory children.

**Conclusion:**

Our data demonstrate prioritization of anticipated benefits and opportunity costs relative to potential harms and procedures in gene therapy trial decision making. Such data inform protocol development, education and advocacy efforts, and informed consent.

## Introduction

Duchenne muscular dystrophy (Duchenne) is a rare X-linked neuromuscular disorder affecting approximately 1 in 5000 newborn males worldwide [1]. Duchenne causes progressive muscle degeneration that becomes apparent around 5 years of age, leading to loss of independent motor function, pulmonary and cardiac complications, and ultimately death [2–4]. Despite variability in the rate of progression among patients with Duchenne, the typical trajectory can be characterized by loss of ambulation, diminishing arm function, and pulmonary decline [5]. Progressive limitations in daily living activities carry significant emotional burden and negatively impact quality of life for people with Duchenne [4, 6, 7] and their caregivers [8–14].

Two non-curative treatments for Duchenne are approved in the United States. One is a corticosteroid therapy that slows muscle degeneration and is approved for all patients [15, 16]. The other is a mutation-specific therapy that received accelerated approval. It is indicated for less than 15% of patients, and clinical efficacy has not yet been established [17–19]. Multiple investigational therapies are currently under development, including gene-replacement therapies [20–22].

Preclinical data from animal models suggest that a truncated version of the *dystrophin* gene introduced through gene transfer can last at least 8 years and may lead to long-term stabilization in muscle function [21, 23, 24]. Three independent trials are currently underway in the United States to establish safety and tolerability of gene therapy in Duchenne patients [25]. In this promising context, we examine interest in participating and the factors important to patients and caregivers when making decisions to participate in clinical trials of gene therapy technologies to treat Duchenne.

Prior research in Duchenne caregivers provides evidence that therapeutic optimism and psychological participation benefits may reduce attention to potential risks and burdens during the trial participation decision-making process; these findings are attributed, in part, to the limited treatment options and to the progressive, fatal nature of the disease [26, 27]. Building on the work of Parent Project Muscular Dystrophy (PPMD) in rare-disease patient-focused drug development [28–30] we used a stated-preference methodology to estimate the relative importance of several plausible risks, burdens, and benefits of gene therapy clinical trials in a hypothetical decision-making process.

## Materials and methods

The study used a community-engaged approach and incorporated an advisory committee comprising three advocacy members, a patient representative, a caregiver representative, an expert clinician, and representatives from the pharmaceutical industry (three representatives each from Pfizer and Solid Biosciences, with rotation of industry representatives over time). The advisory committee provided repeated input on the study aims, instrument, data interpretation, and reporting. Scientific oversight was provided by RTI International/RTI Health Solutions. Community leadership came from the sponsoring organization, Parent Project Muscular Dystrophy (PPMD). As part a larger online survey, we conducted a best-worst scaling (BWS) choice experiment [28] examining factors important in decision making regarding participation in early-phase clinical trials for gene therapy as a treatment for Duchenne muscular dystrophy (Duchenne). The study protocol received IRB review and approval from RTI International’s Committee for the Protection of Human Subjects.

### Participants

PPMD recruited caregivers (parents or legal guardians) of people with Duchenne and adult males with a Duchenne diagnosis through the self-report Duchenne Registry (www.duchenneregistry.org). Four sequential email notices were sent to eligible registry participants. All participants were at least 18 years of age, living in the U.S., and able to read and answer the online survey in English. Eligible caregivers were parents or guardians of a living person, of any age, with Duchenne.

### Study procedures

The online questionnaire was conducted using Qualtrics [31] and was administered from March 1 – April 2, 2018. In addition to the BWS experiment, the online questionnaire also collected demographic information, ambulatory status of the reference person with Duchenne, and two questions related to interest in participating in gene therapy trials. In addition, the questionnaire included a threshold experiment designed to estimate the maximum risk of death that participants would tolerate from gene therapy; the threshold experiment was conducted prior to the BWS experiment. The survey was anchored around a vignette about gene therapy for Duchenne that described a non-curative potential benefit with uncertain but limited durability. Here we report results only from the BWS experiment.

#### BWS experiment

The BWS experiment adhered to an object case design [32]. In this study, we defined 33 choice sets comprising a limited and narrowly defined set of features, or *objects*. The objects represented potential advantages and disadvantages that adults with Duchenne or their caregivers may consider important when deciding whether to enroll in an early-phase gene therapy trial. Participants were shown a sampling of these choice sets and selected the two objects in each set that they would care about the most and the least, respectively, if they were deciding whether to enroll in such a trial. Caregivers who had more than one child with Duchenne were instructed to make these selections with their youngest living child with Duchenne in mind.

We constructed the choice sets from a common set of 11 objects comprising three potential benefit items, two potential risk items, one item about benefit durability, two items about loss of future-treatment or trial options, and three procedural/trial-burden items. These are shown in Table 1. In choosing these objects we drew on our findings from qualitative interviews [33]. Items were selected and refined in collaboration with our multi-stakeholder project advisory committee. Our objective in item selection was that all items would be highly relevant to clinical trial decision making.

**Table 1:** Objects used to construct choice sets for the best-worst choice experiment

Object	Description
Chance of improved muscle function	Data are positive about the chance of maintaining, and maybe improving, muscle function.
Chance of improved heart function	Data are positive about the chance of maintaining, and maybe improving, heart function.
Chance of improved lung function	Data are positive about the chance of maintaining, and maybe improving, lung function.
Benefit lasts about 10 years	Data suggest that gene therapy will last for 10 years. It may be shorter or longer, but no one knows. It is currently not possible to give a second dose of gene therapy. It may be possible in the future, but no one knows.
Chance of being in placebo group	The trial uses a placebo group, where some participants are randomly assigned to a group that gets an inactive (fake) treatment. People who get placebo during the trial ***would be*** eligible for gene therapy in the future.
Lowest dose may be too low for benefit	One of the trial’s goals is to test the right dose of gene therapy. If participants get a dose that is too low to work, they ***will not*** get another chance to use gene therapy.
Two muscle biopsies required	Being in the trial requires 2 muscle biopsies (one from the arm and one from the leg) to test for dystrophin production.
Not eligible for future trials	People who get gene therapy will most likely not be eligible for other clinical trials for the rest of their lives. It may someday be possible, but no one knows.
Limits later use of gene therapies or CRISPR	People who get gene therapy may not be able to use some newer types of gene therapy or gene editing (like CRISPR) for the rest of their lives. It may someday be possible, but no one knows.
Chance of long hospitalization	Data suggest a low risk of needing a long hospitalization of 4 weeks or more to recover from serious side effects.
Chance of death (low risk)	Data suggest a very low risk of death soon after using gene therapy. That risk should be even lower than we showed you in the first survey task.

We asked participant to imagine they or their child had been invited to enroll in an early-phase gene therapy clinical trial for Duchenne. The objects and descriptions reflect the information provided to participants. We explained to participants that references to data in the descriptions referred to evidence collected from animal studies

**Fig. 1:**
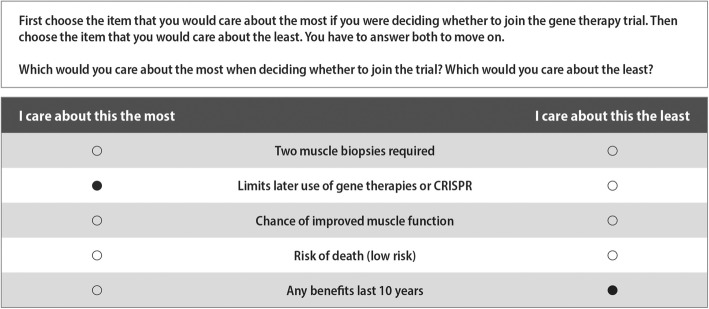
Example best-worst scaling choice task

All participants answered 11 choice questions containing five objects apiece. We used a partially balanced incomplete block design (PBIBD) to create the choice sets used in this study. Participants were randomly assigned to complete one of three versions of the BWS experiment, each of these blocks was made up of 11 distinct choice sets. In the resulting design, each object appeared five times across all 11 sets in each block. The design was partially balanced with variation in the number of times each pair of objects appeared across sets, ranging from 1 to 4 times. The algorithm used to construct the design achieves near-optimal efficiency and our analytical approach produces unbiased estimates even in the absence of perfect balance [34]. In the choice tasks, participants were first asked to select the feature they would care about the most if they were deciding whether to join an early-phase gene therapy clinical trial for Duchenne, followed by the feature they would care about the least (see Fig. 1). Following the random utility model underpinning BWS, here we assume that participants choose the pair of objects in each choice set representing the greatest difference in personal importance as it relates to participating in a gene therapy trial [35].

### Measures

Prior to analysis, we restructured the choice data so that each choice set contributed nine observations per respondent (guided by Flynn et al.) [35]. Five observations from every choice set could be picked as most important and the remaining four objects could be picked as least important. The resulting stacked data structure presumes that, as instructed, participants first chose the most important feature followed by the least important, and is consistent with the imposed constraint that participants were not able to select the same object in a set as both the most and least important feature. With 274 participants in our final analytic sample, 11 choice sets per participant, and 9 objects per set, our final restructured dataset had a total of 27,126 observations.

The dependent variable was an indicator coded 1 whenever the object was selected as either most or least important, and 0 otherwise. The independent variables included a set of object identifiers and interaction terms used to test for differences by mobility and participant subgroup (i.e., adults and caregivers). The object identifiers were represented by ten dummy coded variables indicating which object was available to be selected in each observation. “Chance of improved muscle function” was treated as the reference category and signified in the dataset by observations where the values of all ten object identifiers were 0. Unlike standard dummy coding, observations for the four objects in each choice set that remained to be selected as least important were reversed, taking a value of − 1 instead of + 1 (which was used for objects that could be selected as most important). Reverse coding in this way sets a common scale for the parameter estimates [32]. The interaction terms allowed us to perform a moderation analysis comparing the estimated importance of each gene-therapy trial characteristic among (1) non-ambulatory adults with Duchenne (i.e., those who reported using a wheelchair most or all of the time), (2) caregivers of non-ambulatory children with Duchenne, and (3) caregivers with a child who is ambulatory (i.e., walks with assistance or independently). Our motivation for testing for interaction effects is based on the recognition that these subgroups may have different perspectives on gene-therapy trials that would otherwise be masked in a main-effects only model. Four ambulatory adults with Duchenne completed the BWS experiment, but the small sample size prevented us from fully crossing mobility by participant subgroup. To facilitate interpretation of interaction effects, we did not include these four participants in the analyses reported here. We computed the interaction terms by multiplying each object identifier by a set of dummy variables indicating the segment to which participants belonged. Adults with Duchenne who use a wheelchair were designated the reference group in these interactions.

Best-worst scaling experiments can function well even if participants have little interest or intention to engage in the relevant behavior (i.e., participants are capable of choosing most and least important objects, regardless of relevance). Thus, we asked two questions after the BWS experiment to assess interest, framed as the likelihood of attending an information and screening visit given two scenarios. The first specified a 3-h visit to the patient’s regular neuromuscular clinic with a physical exam and blood draw. The second added additional burden of an 8-h car ride, 2-night hotel stay, and a full-day assessment including muscle function testing. Participants were asked the likelihood they would attend the screening visit. Response options ranged from *not at all likely* to *very likely*.

### Statistical analysis

We analyzed data from our BWS experiment using a respondent-level marginal sequential approach [32, 35] with conditional logistic regression in Stata 15.0 [36]. The marginal sequential approach fits conceptually with the instructions to the respondents to follow a most-then-least selection process [37].

The regression coefficients and corresponding odds ratios from this analysis are estimates of relative importance for the 11 objects. Larger coefficients and odds ratios indicate objects with greater importance to enrollment decisions. We computed robust standard errors that adjust for clustering by participant. Given our interest in exploring subgroup differences in preferences, we used a multistep modeling approach by first estimating a main effects model before adding interaction terms. We conducted Wald tests to compare models at each step, retained only those interaction terms for trial characteristics with importance estimates that significantly differed by at least one subgroup (*P* < .05), and report detailed results from the final model.

## Results

### Participant characteristics

Participant characteristics are presented in Table 2. The recruitment notice was opened by 594 Registry participants; 319 individuals participated in the survey and 278 participants completed the BWS experiment. The response rate is difficult to determine as adults and caregivers may have received the same recruitment notice and/or forwarded the notice to others. As previously described, the four ambulatory adults with Duchenne who completed the BWS experiment are not included in this analysis. Adult participants with Duchenne included in the analysis (*n* = 27) were non-ambulatory and ranged in age from 19 to over 40 years (median = 27). Caregivers (*n* = 247) were 26 to 72 years of age (median = 44). Caregivers reported the age of their youngest living child with Duchenne, which ranged from 1 to older than 40 years (median = 11). The majority of participants (57%) were caregivers of an ambulatory person with Duchenne.

**Table 2:** Participant characteristics by subgroup

Variable	Adults with Duchenne		Caregivers		Total	
	n	%	n	%	n	%
Mobility						
Uses wheelchair most or all the time	27	100.0	91	36.8	118	43.1
Walks with assistance or better	–	–	156	63.2	156	56.9
Sex						
Male	27	100.0	53	21.5	80	29.2
Female	–	–	194	78.5	194	70.8
Race/ethnicity						
White, non-Hispanic	17	63.0	193	78.1	210	76.6
Black, non-Hispanic	0	0.0	4	1.6	4	1.5
Hispanic, all-races	5	18.5	20	8.1	25	9.1
Other, non-Hispanic	3	11.1	27	10.9	30	11.0
Refused	2	7.4	3	1.2	5	1.8
Marital status						
Married or committed relationship	1	3.7	211	85.4	212	77.4
Single	24	88.9	8	3.2	32	11.7
Divorced or separated	0	0.0	27	10.9	27	9.9
Widowed	0	0.0	1	0.4	1	0.4
Refused	2	7.4	0	0.0	2	0.7
Educational attainment						
High school or less	14	51.9	49	19.8	63	23.0
Technical school or associate degree	3	11.1	34	13.8	37	13.5
Bachelor’s degree	3	11.1	94	38.1	97	35.4
Graduate or professional degree	5	18.5	69	27.9	74	27.0
Refused	2	7.4	1	0.4	3	1.1
Annual household income						
Less than $50,000	8	29.6	12	4.9	20	7.3
$24,000 – $50,000	1	3.7	22	8.9	23	8.4
$51,000 – $75,000	1	3.7	30	12.2	31	11.3
$75,001 – $100,000	1	3.7	45	18.2	46	16.8
More than $100,000	3	11.1	110	44.5	113	41.2
Prefer not to answer or refused	13	48.2	28	11.3	41	15.0
Previous clinical trial participation						
Yes	5	18.5	128	51.8	133	48.5
No	22	81.5	119	48.2	141	51.5

*N* = 274. Percentages may not add up to 100% due to rounding

A large majority of participants expressed interest in attending an information and screening visit as a precondition for trial participation. For the less burdensome scenario involving a 3-h visit at the regular clinic, 97% of caregivers reported being very likely or somewhat likely to attend. Among adults with Duchenne, 96% were very likely or somewhat likely to attend. For the second scenario involving an 8-h drive, 2-night stay, and muscle function testing, 89% of caregivers said they were very likely or somewhat likely to go to the visit. Adults with Duchenne reported lower interest, with 70% endorsing very likely or somewhat likely.

### BWS analysis

In the final model (Table 3), the chance of improved lung function among adults with Duchenne served as the reference group for moderation analyses. A joint test of the two remaining interaction terms revealed that preferences for improved lung function differed significantly by subgroup (*P* < .001). Specifically, caregivers of ambulatory children cared significantly less about the chance for improved lung function when deciding to join a gene therapy trial than either adults with Duchenne (*P* < .001) or caregivers whose child uses a wheelchair (*P* < .001). There was no statistical difference between caregivers of children who use a wheelchair relative to adults with Duchenne who use a wheelchair (*P* = .115).

**Table 3:** BWS conditional logistic regression

Object				95% CI		
	B	SE B	OR	LL	UL	*P*
Chance of improved muscle function^a^	0.00	–	1.00	–	–	–
Chance of improved lung function	−0.14	0.18	0.87	0.61	1.25	.450
Limits later use of gene therapy or CRISPR	−0.26	0.12	0.77	0.61	0.98	.033
Chance of improved heart function	−0.28	0.08	0.75	0.65	0.87	<.001
Chance of death (low risk)	−0.84	0.13	0.43	0.34	0.56	<.001
Lowest dose may be too low for benefit	−1.20	0.12	0.30	0.24	0.38	<.001
Become ineligible for future trials	−1.20	0.13	0.30	0.23	0.39	<.001
Benefit lasts about 10 years	−1.32	0.09	0.27	0.23	0.32	<.001
Chance of long hospitalization	−1.75	0.11	0.17	0.14	0.22	<.001
Chance of being in placebo group	−1.98	0.12	0.14	0.11	0.17	<.001
Two muscle biopsies required	−2.49	0.11	0.08	0.07	0.10	<.001
Interaction terms						
Improved lung function × Adults with Duchenne^a^	–	–	–	–	–	–
Improved lung function × Parents of children who walk	−0.94	0.19	0.39	0.27	0.57	<.001
Improved lung function × Parents of children who use a wheelchair	−0.32	0.20	0.72	0.49	1.08	.115

*N* = 274. Wald χ^2^ (12)=741.24, *P* < .001, *R*^*2*^_McFadden’s_ = .13. B = Conditional logit regression coefficient. SE B = Robust standard error of B. OR = Odds ratio. CI = Confidence interval for odds ratios. LL = Lower limit of confidence interval. UL = Upper limit of confidence interval

^a^Reference category

**Fig. 2:**
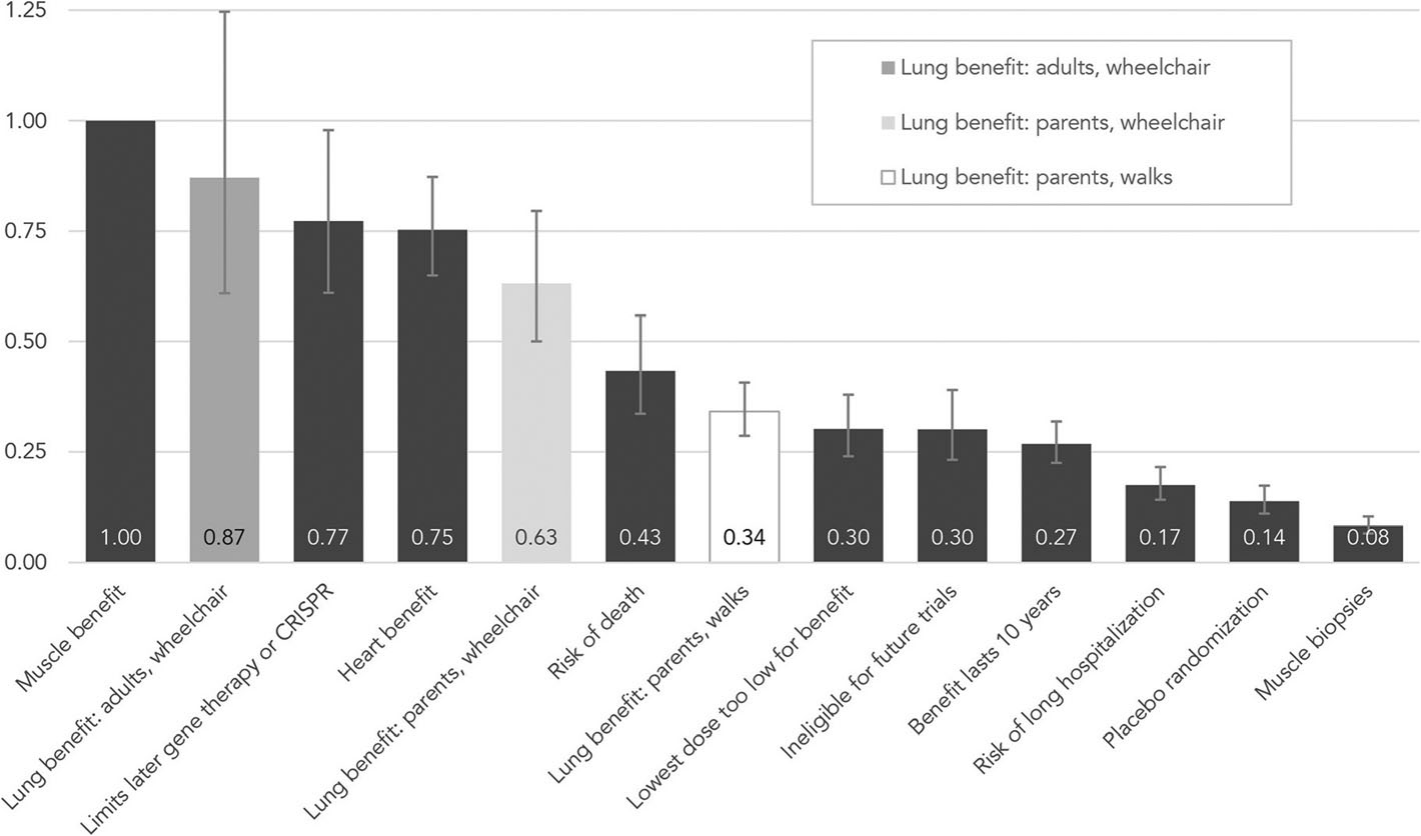
Plot showing relative importance hierarchy for participating in a gene therapy clinical trial. Estimates are odds ratios from the final conditional logit model. The black bars are estimates for which no interaction by participant subgroup was found and represent the average relative importance across all participants. The grey and white bars are importance estimates for “chance of improved lung function” among participants in each subgroup. Error bars represent the 95% confidence intervals for odds ratios. Muscle benefit is the reference category, constrained to equal 1

The remaining regression coefficients in the final model are estimates of relative importance averaged across participant subgroups for the other ten objects. Regardless of participant subgroup, participants cared most about improved muscle function when making decisions about trial participation, which we modeled as the reference category, B = 0 and OR = 1. We plotted the odds ratios and present them in Fig. 2. Odds ratios with non-overlapping confidence intervals are statistically different at the 95% confidence level. Concerns that joining a trial would limit later use of gene transfer or editing (CRISPR) was slightly less important on average than muscle benefit in trial decision making (OR = 0.77; *P* = .033). The possibility that gene therapy would lead to improved heart function was also among the top four features that participants cared about most when considering participating in a trial (OR = 0.75; *P* < .001). In addition to chance of a long hospitalization (OR = 0.17; P < .001), participants cared least about procedural characteristics of clinical trials, like being in a placebo group (OR = 0.14; *P* < .001) or having to have two muscle biopsies (OR = 0.08; *P* < .001).

## Discussion

People with Duchenne have increasing options for clinical trial participation, which may be perceived as providing important opportunities to change the progressive disease course [27]. The first generation of gene therapy is expected to have a non-curative benefit, not dissimilar to other treatments under investigation for Duchenne. This non-curative benefit is coupled with the potential for higher risks for treatment-related morbidity /mortality than in past types of Duchenne clinical trials, as well as “opportunity costs” that are exacerbated by the risks associated with redosing using the same viral vector. Understanding the factors most important in decision making for early phase gene therapy trials allows researchers, sponsors, and advocates to address enrollment challenges, anticipate areas of potential concern in informed consent, and develop targeted educational materials and decision support tools for potential participants.

Caregivers in this study reported high interest, and adults with Duchenne reported high to moderate interest, in attending an information session and eligibility screening if required for trial consideration. These findings support the relevance of the BWS choice tasks to a large proportion of our participants. Overall, participants cared most about the potential benefits of gene therapy when considering gene therapy trial participation. Parents have retrospectively reported the primary importance of the potential benefit in their actual decision-making when enrolling children in other types of Duchenne trials [38] and in a related qualitative study of perceptions of a hypothetical gene therapy trial [33]. Among characteristics included in this study, the chance of maintaining or improving muscle function emerged as most important for all participant subgroups. The chance of heart benefit was also highly important. It was prioritized similarly to concerns that trial participation would disqualify participants from later use of newer gene therapy or gene editing techniques. The disqualification characteristic is the most highly-rated item that differentiates gene therapy from most other types of therapies. The high ranking points to an aversion to losing future therapeutic options. A related gene therapy characteristic, referring to the approximately 10-year duration of benefit without the option for a second dose, fell on the lower end of the importance hierarchy. A prior qualitative study suggests that the lower importance of this feature may reflect optimism that this barrier would be surmounted in 10 years and/or that newer treatments would be available [33].

As we anticipated, caregivers of ambulatory children cared less about the chances of improved lung function than did adults with Duchenne or caregivers of a child using a wheelchair; this finding likely reflects the correlation between pulmonary and ambulatory decline. Temporal discounting may explain the result [39], where the threat to pulmonary health is less immediate for caregivers with a child who is still ambulatory. Thus, we would expect lung-function benefits to become more important as Duchenne progresses.

Procedural trial characteristics were lowest in the importance hierarchy. Participants cared least about two required muscle biopsies. The possibility of assignment to a placebo group was only somewhat more important than the need for muscle biopsies, which diverges from prior results highlighting parental concern about placebo randomization [27, 40]. These findings may represent changing priorities over time or may reflect priorities specific to gene therapy (i.e., for gene therapy, the use of muscle biopsies may be perceived as more acceptable than when used in non-gene-therapy trials; for gene therapy the randomization to placebo equates to maintaining eligibility for later therapies and trials, thus adding a ‘silver lining’). This finding may also reflect one of the benefits of BWS experiments, especially when the objects originate from qualitative research and are selected to be valued by participants. The use of BWS to quantify prioritization among highly-relevant items rather than the use of a scale with Likert-type responses may reduce the chance of skewed data and poor discriminative ability.

Other characteristics of gene therapy—risk of death, ineligibility for future trials, and insufficient dose for benefit—fell into the middle range in terms of influence on decision making. It is important to interpret the relative importance of the risk of death in light of the larger questionnaire that subjects completed; prior to answering the BWS experiment, participants completed a threshold experiment to determine their maximum acceptable risk. The placement of the BWS after the threshold was the primary reason we did not include a quantitative risk estimate in the BWS exercise.

These results should be interpreted with the following limitations in mind. We recruited our patient and caregiver samples through the PPMD network, and the values and preferences expressed in this study may not represent the larger global Duchenne community. Also, respondents may have forwarded the recruitment email, which may have led to non-independence in our final sample. We chose to use an anonymous survey to protect our rare-disease affected participants’ privacy and confidentiality. The resulting limitation is that we cannot identify caregiver/affected adult pairs or evaluate their concordance. In addition, the hypothetical questions will not fully replicate the experience of making decisions about clinical trial participation. Finally, our sample of adult patients with Duchenne was small.

## Conclusion

In the first study of this nature, our findings offer insight into how caregivers of children and adults with Duchenne and adults living with Duchenne prioritize the anticipated benefits, harms, burden, and opportunity costs when deciding whether to join gene therapy clinical trials. Preference studies such as these provide relevant stakeholders with quantifiable evidence to inform the development of emerging therapies, which is a central component of patient-focused drug development.

## Abbreviations

BWS: Best-Worst Scaling
CRISPR: Clustered regularly-interspaced short palindromic repeats
PBIBD: Partially balanced incomplete block design
PPMD: Parent Project Muscular Dystrophy
